# Knockdown of the *Plasmodium falciparum* SURFIN4.1 antigen leads to an increase of its cognate transcript

**DOI:** 10.1371/journal.pone.0183129

**Published:** 2017-08-11

**Authors:** Tatiane Macedo-Silva, Rosana Beatriz Duque Araujo, Kamila Anna Meissner, Wesley Luzetti Fotoran, Márcia Melo Medeiros, Mauro Ferreira de Azevedo, Gerhard Wunderlich

**Affiliations:** Department of Parasitology, Institute for Biomedical Sciences, University of São Paulo, Avenida Professor Lineu Prestes, 1374, São Paulo, Brazil; Kobenhavns Universitet, DENMARK

## Abstract

The genome of the malaria parasite *Plasmodium falciparum* contains the *surf* gene family which encodes large transmembrane proteins of unknown function. While some *surf* alleles appear to be expressed in sexual stages, others occur in asexual blood stage forms and may be associated to virulence-associated processes and undergo transcriptional switching. We accessed the transcription of *surf* genes along multiple invasions by real time PCR. Based on the observation of persistent expression of gene *surf*4.1, we created a parasite line which expresses a conditionally destabilized SURFIN4.1 protein. Upon destabilization of the protein, no interference of parasite growth or morphological changes were detected. However, we observed a strong increase in the transcript quantities of *surf*4.1 and sometimes of other *surf* genes in knocked-down parasites. While this effect was reversible when SURFIN4.1 was stabilized again after a few days of destabilization, longer destabilization periods resulted in a transcriptional switch away from *surf*4.1. When we tested if a longer transcript half-life was responsible for increased transcript detection in SURFIN4.1 knocked-down parasites, no alteration was found compared to control parasite lines. This suggests a specific feedback of the expressed SURFIN protein to its transcript pointing to a novel type of regulation, inedited in *Plasmodium*.

## Introduction

The human malaria parasite *Plasmodium falciparum* possesses a number of variant gene families which encode virulence associated proteins. The most prominent variant gene family consists of the *var* genes [1–3] which encode huge erythrocyte-surface exported antigens termed *P*. *falciparum* erythrocyte membrane protein 1 (PfEMP1) [4]. Versions of PfEMP1 mediate the cytoadherence to endothelial host receptors or receptors on the red blood cell (RBC, for a review, see [5]). The specificity of PfEMP1-receptor interaction may influence the clinical evolution of malaria experienced by infected individuals [6,7]. In order to efficiently evade the immune system, *P*. *falciparum* developed a strict control over *var* gene expression. In fact, only one [8,9] or a few [10] from the 50–60 different *var* alleles per haploid genome are expressed. This is achieved by a strict control of transcriptional activation and silencing of *var* promoters. Multiple factors are involved in this process such as sequence elements in the promoter [11,12] or the *var* intron [13,14] itself, non-coding antisense RNAs [15,16], translational attenuation of *var* transcripts [17,18] and a number of chromatin modifying factors. These are histone deacetylases [19–21], histone methyltransferases [22,23] and Heterochromatin Protein 1 [24,25]. There is also a specific RNAseII which seems to control at least a subset of *var* genes [26]. Recently, specific ncRNAs were described that exert an effect on *var* transcription [27]. Transcriptional switching may occur dependent on the host's response. Accordingly, the observed PfEMP1 switching rate *in vitro* was initially estimated to 2.4% [28] but in natural or experimental infections it can be much higher [29–31]. Very few data exist regarding the regulation of other variant gene families such as *rif*, *stevor* or PfTM-2 genes of which at least *var* and *rif* and perhaps *stevor* seem to be somehow co-regulated [32]. *Rif* genes are transcriptionally controlled since there are only a few *rif* genes transcribed in parasites cultures [33]. Their transcription seems to switch rapidly [34] apparently orchestrated by some of the chromatin modifications which control *var* gene locus activity [35,36]. *Stevor* and PfTM-2 genes do also switch [37]. Nothing so far is known about the members of the much smaller *surf* gene family which encode huge proteins with a N-terminal cystein-rich region, a variable ectodomain, a transmembrane domain and specific tryptophan-rich internal domains [38]. Allelic polymorphism—although much less than observed for *var* genes—is encountered mostly in the variable ectodomain [39,40]. Interestingly, there is a related gene family in *P*. *vivax* termed the PvSTP1 family [41], and STP1 orthologues are expanded in *P*. *malariae* and *P*. *ovale* [42]. The encoded antigens, SURFINs, do not possess a discernible PEXEL element [43] for export into the infected erythrocyte and were found associated to the merozoite surface and/or perhaps the infected RBC membrane [38,44]. Until now, only one surf gene was knocked out and no apparent phenotype was observed [45]. Herein, we set out to monitor the transcription mode of *surf* genes along multiple generations by real time qPCR and we observed an almost constitutive expression of SURFIN4.1 and low quantities of other *surf* transcripts. In order to test its function for the survival of the parasite we produced parasite lines which had a GFP tag and a destabilizing domain integrated in SURFIN4.1, turning the protein regulatable.

## Results

### *Surf* transcription changes over time but *surf*4.1 is stably transcribed

**Fig 1:**
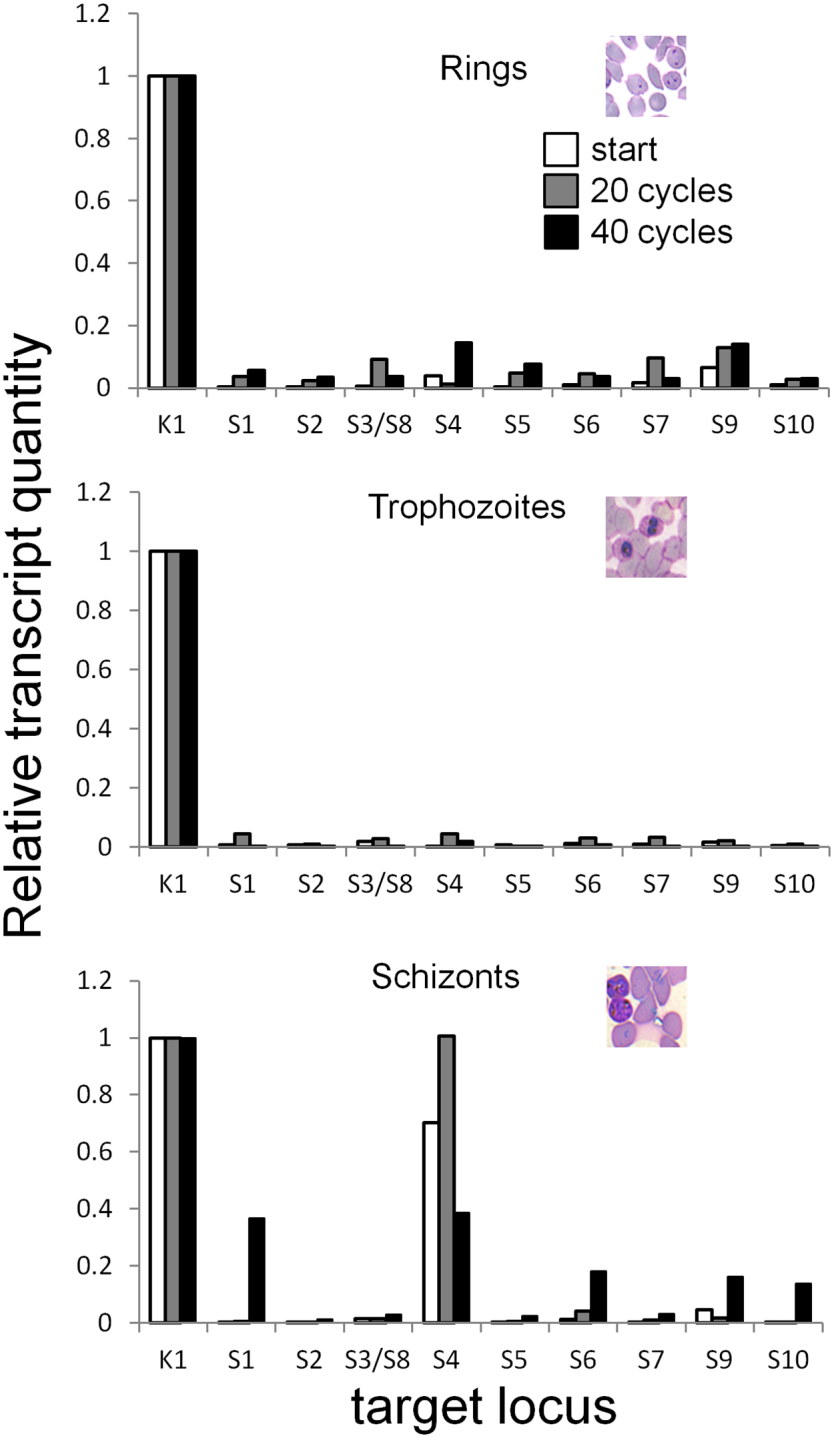
Real time PCR analysis of *surf* transcription in continuous cultures of *P*. *falciparum* NF54. The relative transcript levels were calculated using seryl-tRNA ligase (PF3D7_0717700) as endogenous control (indicated as “K1”). Two cycles before retrieving RNA, parasites were synchronized twice as described and the different stages harvested at identical hours after the final sorbitol lysis. The differently colored bars show the results for 0 (white bars), 20 (grey bars) and 40 reinvasions (black bars). The X axis shows the results for each individual *surf* gene (nomenclature see S1 Table). See Fig A in S1 File for similarly obtained results using the 3D7 strain.

One feature of virulence-associated antigen expression is the sequential expression of genes encoding these antigens, such as happens in the *var* gene family [28] or the *rif* gene family [34]. We monitored differences in *surf* gene transcription in the NF54 isolate during a total of 40 reinvasions and observed that almost all *surf* genes are virtually silenced and only the *surf*4.1 (herein termed *surf*4) transcript was present in schizont stage parasites throughout the experiment (Fig 1). When measuring *surf* transcripts in the NF54 clone 3D7 by using the same approach, similar results were obtained. However, another *surf* transcript (*surf*9) was present at some time points in ring and trophozoite stages (Fig A in S1 File). The somehow continuous expression of SURFIN4.1 suggests a biological role for this protein.

### SURFIN4.1 can be genetically tagged and knocked down in blood stage parasites without loss of viability

**Fig 2:**
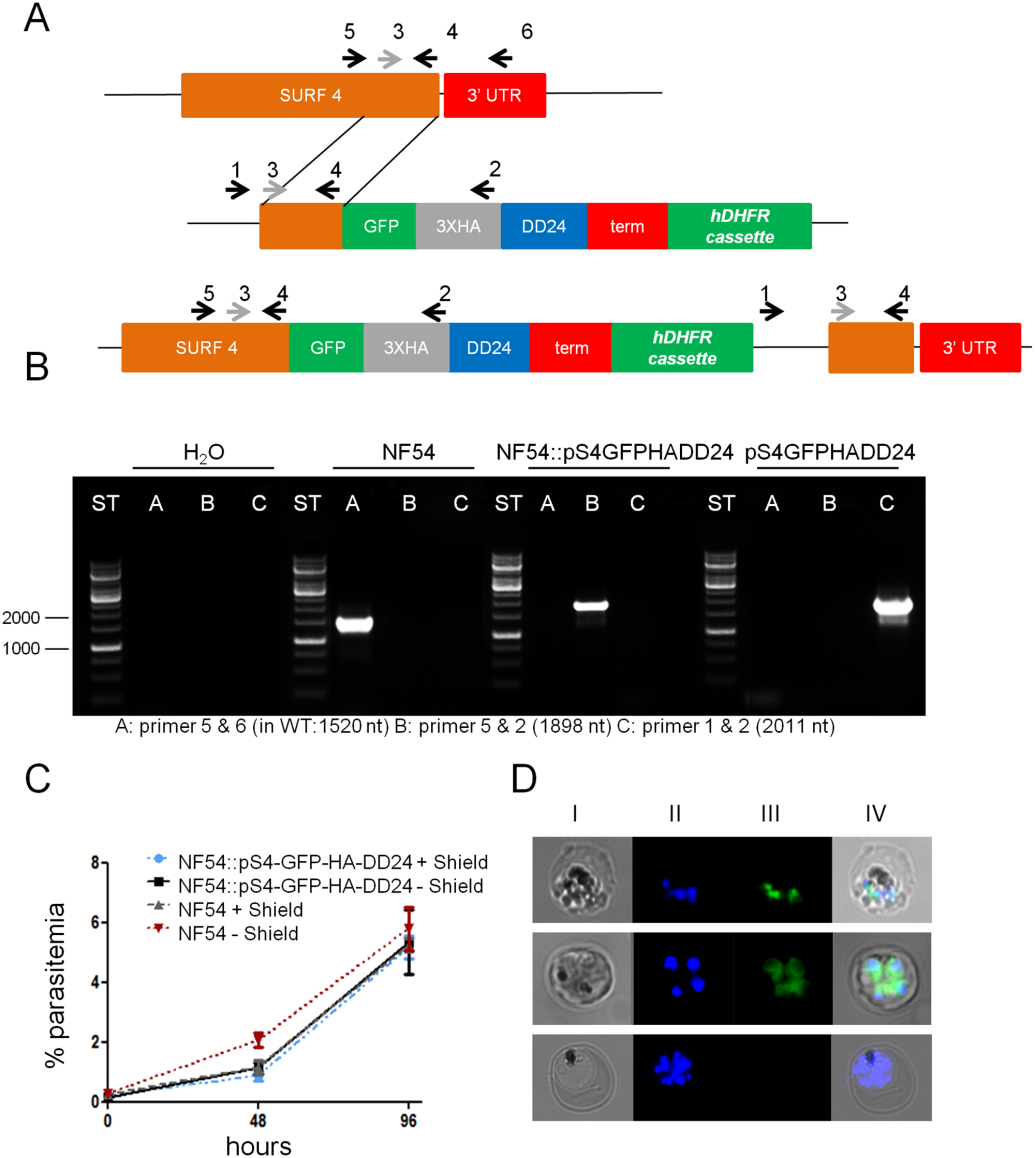
Construction of the knockin plasmids and transfectant clone classification by PCR. In **A**, the proposed model for single crossover recombination of the plasmid pS4-GFP-HA-DD24 is shown. The arrows indicate the localization of oligonucleotides used for PCR. Oligos 3 and 4 amplify the homology region used for the construct. In **B**, PCR results using genomic DNA of the recombinant parasite clone (NF54::pS4GFPHADD24) and controls (NF54 genomic DNA, transfection plasmid pS4GFPHADD24 and water) employed in the experiments are shown. The primer combinations used in PCRs are indicated below the gel picture. On the left, sizes of the DNA molecular weight standard (in base pairs). In **C**, the negligible influence of Shield-1 on the growth of wildtype NF54 or transfectant cultures is shown. In **D**, Fluorescence microscopy with a schizont from the parasite line NF54::pS4GFPHA (upper painels) and NF54::pS4GFPHADD24 with (middle painels) and without 1 μM Shield-1 (lower painel): **I**, bright field, **II**, nuclear staining using DAPI, **III**, GFP-tagged SURFIN4.1 and **IV**, overlay of **I** to **III**.

**Fig 3:**
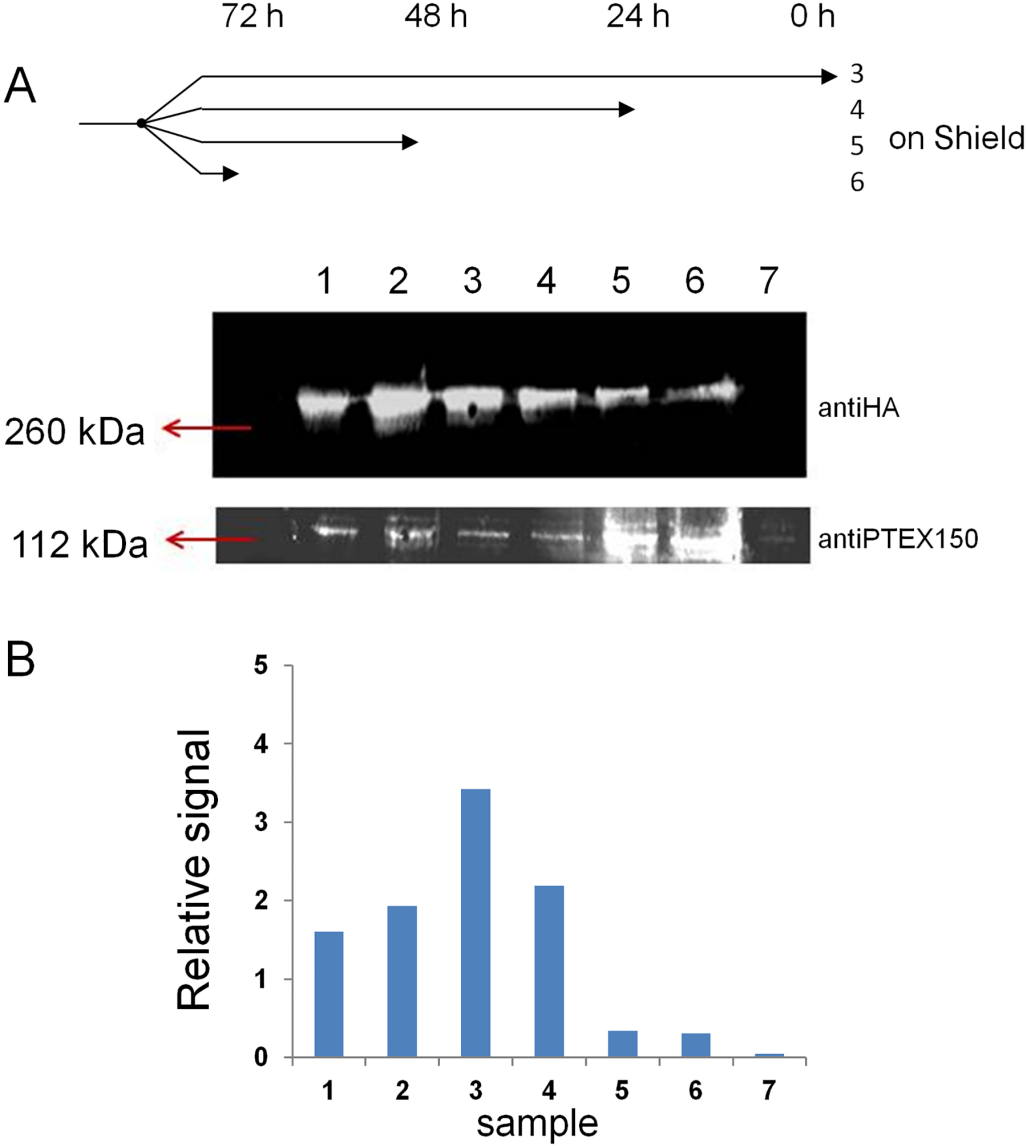
Removal of Shield-1 efficiently knocks down tagged SURFIN4.1. **A**: Outline of the Shield-1 treatment applied to cultures analyzed in **B**. Sample IDs (3 to 6) are indicated on the right side. **B**: Western blot of NF54::pS4-GFP-HA and NF54::pS4-GFP-HA-DD24 in schizont stage and in the presence and absence of Shield-1. In 1 and 2, protein extracts of NF54::pS4-GFP-HA in the presence or absence of 1 μM Shield-1 for 48 h, detected with anti-HA (upper panel, 260 kDa) and antiPTEX150 (lower panel, 112 kDa). Samples 3 to 6 were prepared from one initial synchronized culture on Shield-1 which was split in four aliquots. Then, individual cultures had their Shield-1 removed at given time points. Consequently, sample 3 consisted of protein extracts of NF54::pS4-GFP-HA-DD24 parasites in the continuous presence of 1 μM Shield-1. In 4, Shield-1 was removed 24 h before harvest, in 5, Shield-1 removal 48 h before harvest and in 6, 72 h before harvest. Samples 3 to 6 were harvested at once, meaning that the difference between sample 3 and 6 is that sample 6 was cultivated 72 h without Shield-1. In 7, wild type NF54 parasites showing no detectable signal in 260 kDa (no HA tag present) and a weak signal for PTEX150. In **B**, densitometry analysis of the observed signals using ImageJ, normalizing to the PTEX150 signal.

In order to examine a potential role in blood stage parasites, we created two NF54 parasite lines which had their *surf*4 gene genetically tagged with either a GFP-HA tag or a GFP-HA-DD24 tag (Fig 2). The latter is supposed to turn the protein regulatable through the addition or removal of the small ligand Shield-1 [46,47]. Repeatedly cycled and finally cloned transfectant parasite lines containing an integrated version of the plasmid pSURF4-GFP-HA-DD24 were PCR tested and no amplification product was obtained with an oligo pair which detects episomal, non-integrated constructs (Fig 2). The parasite line containing an integrated version of the plasmid without the DD24 domain was also cloned and all parasites showed green fluorescence in cytometry analysis. Fluorescent microscopy revealed a merozoite-associated pattern in late schizonts and an absence of any discernible signal at the infected red blood cell surface (Fig 2). We then tested if the decrease of SURFIN4.1 had an effect on the viability of parasites in which SURFIN4-GFP-HA-DD24 was destabilized through the removal of Shield-1. No profound effect on the parasite growth was observed and parasites showed only a slight decrease in viability when grown in the presence of Shield-1, an effect visualized also by others (Fig 2, [47]). No increase in gametocyte production or abnormal parasite forms was observed and no extension or decrease of the blood stage cycle duration was evidenced. It was then checked if there was indeed a loss of SURFIN4.1-GFP-HA-DD24 upon Shield-1 removal and this was done first fluorescence microscopy. Removal of Shield-1 led to a complete absence of fluorescence in schizont parasites (Fig 2D, bottom). We then quantified the knockdown efficiency by Western blots with antiHA antibodies and the control antiserum against PTEX150, a component of the secretion machinery of the parasite [48]. As shown in Fig 3, there was a significant decrease of the fusion SURFIN4.1-GFP-HA-DD24 upon removal of Shield-1. Importantly, the relative quantity of the control antigen PTEX150 was not profoundly altered when testing SURFIN4.1-GFP-HA parasites in parallel, nor was the signal of SURFIN4.1-GFP-HA. When calculating the signal intensities using ImageJ, a knockdown of over 90% for the destabilized fusion protein after 48 h was achieved which no longer decreased after removal of Shield-1 for 72 h when compared to the initial, Shield-1-treated parasites. Therefore, it seems that SURFIN4.1 is expressed in blood stage schizonts but its role is not of immediate importance for survival or growth of the parasite. Alternatively, the residual SURFIN4.1-GFP-HA-DD24 is still able to exert its biological function in the parasite or else there was a compensation of other SURFINs of expression increased upon knockdown of SURFIN4.1.

### Knockdown of SURFIN4.1-GFP-HA-DD24 leads to increased *surf4*.*1* transcript levels

**Fig 4:**
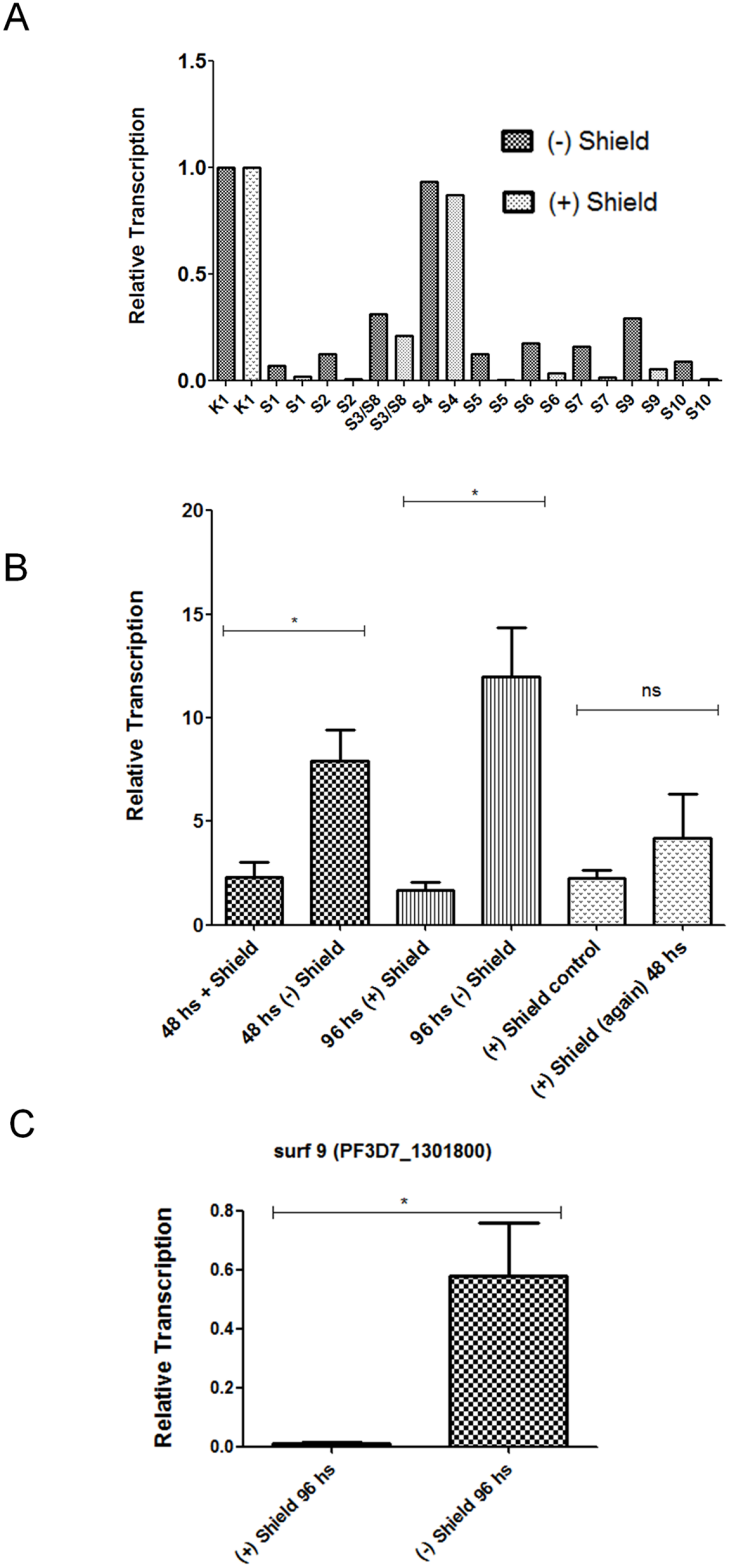
Knockdown of SURFIN4.1 leads to the increase of steady state levels of *surf*4 transcripts and perhaps others. In **A**, no profound effect on the *surf* transcript quantities is seen when 1 μM Shield-1 is applied to NF54 cultures for 48 h (measured by RT-qPCR as described). K1 is the endogenous control (tRNA-seryl ligase transcript, done for each time point and RNA). In **B**, effect of the removal of Shield-1 on relative *surf*4 transcript levels in NF54::pS4-GFP-HA-DD24 parasites. For this, a starting culture NF54::pS4-GFP-HA-DD24 kept under Shield-1 treatment was split into parallel cultures with or without shield-1 for a maximum of 96 h after which shield-1 was re-added in the cultures without shield-1. This experiment was repeated three times and the average values for relative transcript abundance is shown. Differences were statistically evaluated using Student’s T test and an asterisk indicates significant differences (p<0.05). The *surf4* transcript levels increased upon removal of shield-1 and returned to previous values when shield-1 is added back. In **C**, the difference in the transcript quantity of *surf*9 after protein knockdown of SURF4.1 in cDNA samples from **B** at 96 h on/off shield-1 are shown. Note that while *surf4* showed more transcript at 96 h without Shield-1 (as shown in **B**, coinciding with decreased SURF4.1 protein, compare with Fig 3), the quantity of the *surf9* transcript also reproducibly increased.

In variant gene families, there is often a transcriptional switch that causes the silencing of one allele and the activation of another allele, such as in the *var* gene family. Accordingly, we were interested in the question if the lack of phenotype in the SURFIN4.1 knockdown was caused by the increased expression of another SURFIN which then functionally substituted SURFIN4.1. As there are yet no specific antibodies available, we monitored instead the *surf* transcripts in knocked-down parasites as a surrogate marker (Fig 4). This was done by retrieving Shield-1 for different time intervals, maintaining the original Shield-1 treated culture in parallel. Importantly, wild type parasites treated or not with Shield-1 did not greatly differ in their quantity of *surf*4.1 transcripts, meaning that Shield-1 itself does not interfere in *surf* transcript quantities (Fig 4A), although a slight increase in all *surf* transcripts could be observed in the wild type NF54 line when Shield is retrieved from the culture. This was most visible in *surf* transcripts which are virtually not transcribed in the presence of Shield-1 such as *surf*2, 5, 8 and 9. In contrast, when we measured the transcripts of transgenic knocked-down parasites, we observed a strong and reproducible increase of the *surf*4.1 transcript (Fig 4B). This experiment was independently repeated three times with similar results. Additionally, other *surf* transcripts such as *surf*9 or *surf*3/8 were also increased after the SURF4.1 knockdown (Fig B in S1 File). If the observed effect is indeed a kind of regulation, then this phenomenon is likely to be reversible. Accordingly, transcript quantities should decrease when SURFIN4.1-GFP-HA-DD24 is re-stabilized after a period of destabilization. In agreement with this, the *surf*4.1 transcript decreased to lower levels when Shield-1 was added back for 48 h. The quantities of *surf*4.1 transcripts after re-stabilization varied between different experiments but were always much lower than in parasites with SURFIN4.1GFP-HA-DD24 destabilized for two or three reinvasion cycles (Fig 4B). This suggests that the intact SURFIN4.1-GFP-HA-DD24 protein has somehow a modulating effect on its cognate transcript and perhaps also on other *surf* transcripts. Of note, cultures kept in parallel on Shield-1 during the course of the experiment show similar *surf*4.1 transcript levels at the end of the experiment when compared to parasites at the start of the experiment (4B, compare bars “control +Shield-1” on the right). Interestingly, the steady state level of the *surf9* is also reproducibly increased upon knockdown of SURFIN4.1 in NF54 (Fig 4C and Fig B in S1 File). However, given that the absence of Shield-1 in wildtype NF54 also led to an increase to *surf*9 transcripts, it is not ruled out that this is at least partially a Shield-induced and non-specific effect.

**Fig 5:**
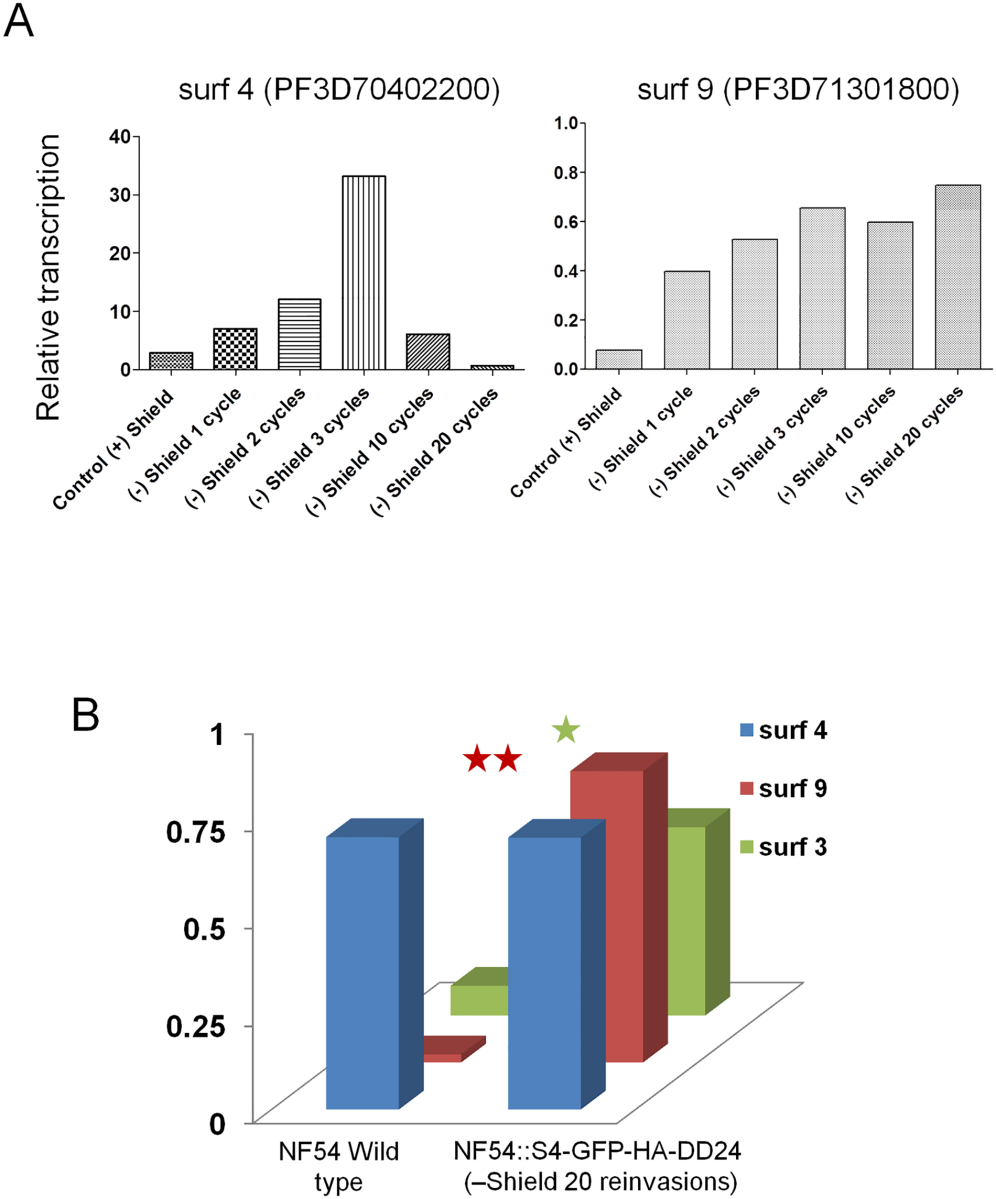
Effect of long term depletion of Shield-1 in NF54::pS4-GFP-HA-DD24 cultures on *surf* gene transcription. In **A**, dynamics of the *surf*4 and *surf*9 relative transcript quantity at given time points after removal and re-addition of Shield-1. Note that surf9 transcripts steadily increase while *surf*4 transcripts decrease after continued SURF4.1 protein knockdown. Results from one representative experiment of three is shown. In **B**, 3D panel directly comparing NF54 wildtype parasite transcription of *surf*4, *surf*3/8 and *surf*9 versus NF54::pSURF4-GFP-HA-DD24 knocked down parasites after outgrowth of 20 cycles without Shield-1 in three independent experiments. One asterisk means significant differences in transcript quantities (p = 0.0043, Student’s T test), two asterisks mean highly significant differences (p < 0.0001, Student’s T test).

We then tested if the observed increase in *surf*4.1 transcripts is maintained over longer periods of Shield-1 removal and knockdown of SURFIN4.1. For this, parasites were grown for 10 and 20 reinvasions without Shield-1. As shown in Fig 5, *surf*4.1 transcript levels decreased to quantities even lower than on-shield parasites with the difference that two other *surf* transcripts were observed in significantly higher amounts, namely *surf*9 and *surf*3/8. It is possible that a transcriptional switch occurred in these parasites, silencing the *surf*4.1 locus in most of the parasites while other SURFINs are expressed in parasites instead of unstable SURFIN4.1.

### Increased *surf*4.1 transcript half-life is not responsible for the observed transcript increase upon SURFIN4.1 knockdown

**Fig 6:**
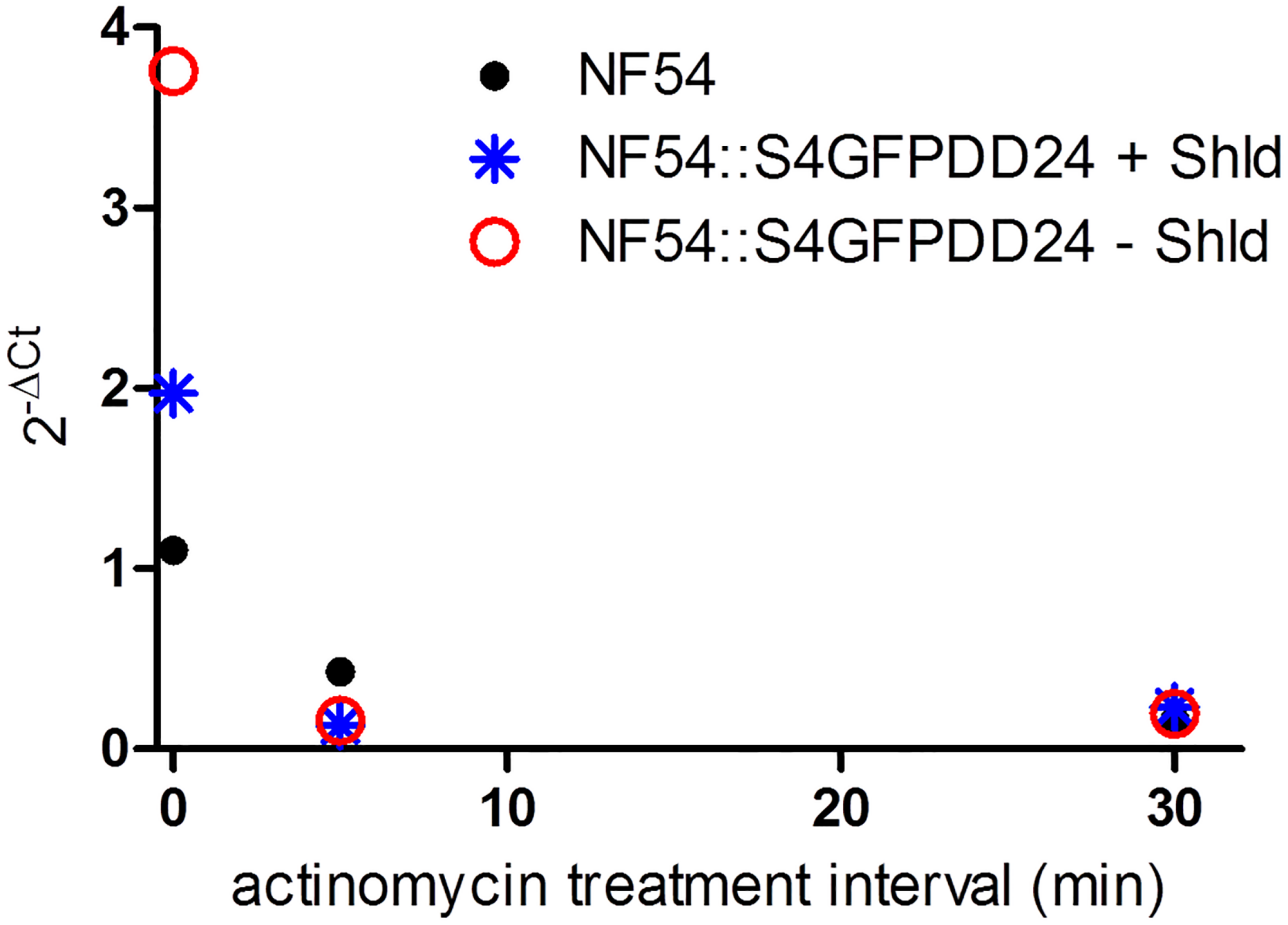
Protein destabilization does not influence the half-life of the *surf*4 message in actinomycin D treated knocked down or normal parasites. Parasites treated for different intervals with actinomycin D were harvested and had their RNA purified and same quantities of total RNAs were reverse transcribed and analyzed by qPCR. For each timepoint and sample, the 2^-ΔCt^ were calculated from the internal control PF3D7_0717700 and the *surf*4.1 transcript. In case of identical decay of transcripts, similar 2^-ΔCt^ values would be expected while longer half-lifes would lead to increased values at longer incubation times. Accordingly, shorter half-lifes of transcripts compared to the internal control PF3D7_0717700 would lead to decreasing values after longer incubation with actinomycin D. Note that in all parasites decreasing 2^-ΔCt^ are observed which is consistent with relatively shorter half-life of the surf4.1 transcripts compared to PF3D7_0717700 transcripts.

In order to explore if increased *surf* promoter activity or transcript accumulation due to altered *surf*4.1 mRNA decay is responsible for the observed phenomenon of transcript increase, we submitted Shield-1 treated or untreated parasites (augmented *surf*4.1 transcript presence) to actinomycin D treatment. If *surf*4.1 transcripts show a prolonged half-life when parasites are submitted to SURFIN4.1 knockdown, this effect should be measurable after actinomycin D treatment. Actinomycin D inhibits RNA polymerase 2, turning the decay of mRNAs measurable after different time intervals of treatment. As shown in Fig 6, we observed a rapid decay of the *surf*4.1 transcript in relation to the internal control t-seryl ligase. As the decay was observed in wildtype NF54 and also transfectant parasites with or without shield-1, we conclude that the *surf*4.1 is more unstable than the housekeeping t-seryl ligase transcript and that protein destabilization does not alter the *surf*4.1 transcript stability. These results indicate that the higher quantities of *surf*4.1 transcripts in SURFIN4.1-knocked down parasites is most likely due to higher promoter activity than decreased mRNA degradation.

## Discussion

An important feature of virulence associated surface-exposed antigens is that they either i) must be very little immunogenic or short lived (such as the novel invasion related antigen PfRH5, [49]), ii) show high variability such as many plasmodial merozoite surface proteins, or iii) use antigenic variation and sequential expression of antigens such as reported for the *var* genes in *P*. *falciparum*. In this work, we showed that the strain NF54 and also its clone 3D7 showed a predominant and stable expression over time of one *surf* gene out of 10 alleles present in the NF54/3D7 genome. This observation turns unlikely a role in antigenic variation and immune escape. A number of studies showed that the transcriptional switching of variant genes such as *var* genes is different in patient isolates when compared to *in vitro* cultures [29,30]. This may also be the case for *surf* genes although this point has never been properly addressed and perhaps there is a differential expression of SURFINs during single blood stage infections. The published transcriptomes of the 3D7 strain in PlasmoDB indicate that a number of *surf* genes are probably transcribed in insect stage parasites, such as ookinetes or oocysts. However, PlasmoDB information is a snapshot and does not display (the extent of) transcriptional switching during several reinvasions. Our data are in concordance with the transcriptome data confirming that the most consistent transcript is indeed from the *surf*4.1 locus and is produced in schizont stages.

The consistent expression indicates a biological role for SURFIN4.1 in the blood stage forms. The *surf*4.1 gene is unusual for the fact that it is annotated as a pseudogene and the NF54 lineage used herein indeed showed numerous stop codons for all possible reading frames upstream from the fragment used for genetic tagging, identical to the strain annotated in PlasmoDB. Even after splicing as predicted in PlasmoDB, at least one stop codon persists in the predicted mRNA sequence of *surf*4.1. Given that we were able to produce SURFIN4.1 with C-terminal GFP-HA or GFP-HA-DD24 tags, it seems that an additional editing event occurred. This issue is actually being addressed in more detail, and other authors also already documented uncommon splicing variants of this gene [50,51].

In order to attribute a biological function for SURFIN4.1, parasites with SURFIN4.1 genetically fused to GFP and HA were made. We also created a parasite lineage where SURFIN4.1 was fused to GFP, HA and DD24. When the stabilizing reagent Shield-1 was removed from the latter parasite line, a significant decrease of SURFIN4.1-GFP-HA-DD24 was observed. Importantly, this decrease was specific since simultaneously treated SURFIN4.1-GFP-HA expressing parasites neither showed more or less of the corresponding protein in the presence or absence of Shield-1, nor did the relation PTEX150/SURFIN4.1-GFP-HA change. The achieved knockdown was estimated to around 90% and this is slightly more than what was observed by Azevedo and colleagues [47] for an adjustable GFP-luciferase construct. However, no significant effect on growth was found in knocked-down parasites. At least two possibilities may explain this observation. First, SURFIN4.1 exerts a function that is not necessary in *in vitro* situations. Second, SURFINs are somehow important—perhaps in invasion processes such as suggested by Winter and colleagues [38]—and knockdown of one SURFIN leads to the swift expression of another SURFIN compensating for the loss of function of the formerly expressed SURFIN allele. To test the second option, we asked if the knockdown of *surf*4.1 had any effect on the other SURFINs/*surf* transcripts. In the absence of specific antibodies to any of the SURFINs we measured the steady state levels of *surf* transcripts in knocked down and control parasites. Unexpectedly, the decrease of the SURFIN4.1 protein resulted in a strong, significant and reproducible increase of the *surf*4.1 transcript. To our knowledge, there is only one hint that shows that the directed depletion/blockade of a protein in *Plasmodium* may lead to an up-regulation of the transcript encoding it. In this specific case, Zhang and Rathod found that the plasmodial dihydrofolate reductase binds to its cognate mRNA, blocking further translation of the RNA even when the enzyme is blocked by inhibitors—contrary to what happens to the human dihydrofolate reductase [52]. In the case of SURFIN4.1, a complementary effect may take place in that less SURFIN4.1 led to increased *surf*4.1 mRNA levels. There are many examples of metabolite-regulated feedback mechanisms which resulted in an increase of transcripts of a metabolic enzyme, such as the regulation of carotenoid biosynthesis in plants (reviewed in [53]). In one report for *Plasmodium*, Cassera and colleagues showed only insignificant increases of transcripts encoding enzymes of the methylerythritol phosphate (MEP) pathway upon addition of fosmidomycin, an inhibitor of the MEP pathway. The addition of chloroquine to cultures also did not result in any significant change in PfCRT expression [54]. However, all these reports cannot be compared directly since in these cases the targeted proteins—metabolic enzymes—are blocked but not destabilized.

No biological role has been attributed to SURFIN and so far, no information is available if SURFINs have some metabolic functions which—when abolished—would then result in the depletion of a metabolic end product. Therefore, it is speculative to assume that the lack of the hypothetic metabolite would then provoke the increase of the cognate (or an equivalent) SURFIN transcript. If so, this might happen either by interacting with hypothetical secondary RNA structures or repressor proteins such as it is the case in the bacterial model (Tryptophan synthesis or tetracycline repressor) or through more complicated mechanisms by influencing the epigenome such as happens in the regulation of the molecular clock in human cells (reviewed in [55]).

In order to address the question if the accumulation of *surf*4.1 transcripts in SURFIN4.1 knockdowns was in fact occurring due to an increased stability of *surf*4.1 mRNAs, perhaps by their increased recruiting to ribosomes somehow provoked by the rapid decay of its end product SURFIN4.1, we assessed the mRNA decay of transcripts in wild type, Shield-depleted and Shield-treated parasites. Interestingly, the half-life of the *surf*4.1 transcript is much shorter than of the t-seryl ligase transcript in our assays, in contrast to previous microarray data [56]. It is possible that the different techniques (qPCR versus microarray) are responsible for this discrepancy. Nevertheless, no difference in the relative *surf*4.1 message half-life could be observed comparing either wild type parasites or Shield-treated or untreated parasites. This suggests that the control of *surf*4.1 transcript levels occurs most probably at the transcriptional level. It remains unclear how this may happen in *Plasmodium* parasites. One mechanism of feedback involving the unfolded protein response via *perk* in the endoplasmic reticulum, eIF2alpha phosphorylation and ATF4 was suggested for the selection of odorant receptors in olfactory cells of mammals [57]. This pathway also includes the histone modifier LSD-1. Although some of the involved factors involved in odorant receptor expression control can be identified in the plasmodial genome sequence, one would expect huge differences in the way how this control is exerted. For example, it is clear that *surf* transcription can change during multiple reinvasions, while odorant receptors in neurons never change once the neuron is maturated and one odorant receptor is successfully expressed.

If chromatin modification is involved in differential expression of SURFIN4.1 in *Plasmodium*, a fine mapping of chromatin modifications including several lysines at histone 3 would provide an answer to the question if they contribute this phenomenon in SURFIN4.1 knocked down or normally expressing parasites. Notably, chromatin at the *surf* genes does not contain the histone marks found in other variant gene families [58].

Taken together, it appears that *P*. *falciparum* employs an unusual pathway of gene expression control in *surf* genes which differs from that found for the regulation of *var* genes. Further studies involving in-depth analysis of histone modifications at differentially expressed *surf* loci and perhaps proteomic characterization of proteins associated to SURFIN4-GFP-HA may reveal players of a novel pathway of expression control in this deadly parasite.

## Experimental procedures

### Parasite culture and transfection

*Plasmodium falciparum* lineage NF54 [59], kindly provided by Mats Wahlgren (Karolinska Institutet, Sweden) and lineage 3D7 (MR4/ATCC, a clone of NF54), were used throughout the experiments. Blood stage parasites were maintained in RPMI supplemented with 0,23% NaHCO_3_, 0.5% Albumax 1 (Gibco, Rockville MD) and human B+ erythrocytes in a defined gas mixture (90% N_2_, 5% O_2_ and 5% CO_2_) or in candle jars as described earlier [60]. Human erythrocytes were obtained from voluntary blood donors at the São Paulo Medicine Faculty Blood center (Hemocentro) and this aspect of the study had ethical clearance from the local Committee for Experiments involving human material at ICB-USP (Protocol number 847/2017). The synchronization of parasites was done by plasmagel flotation [61] of mature trophozoites followed by sorbitol lysis [62] of ring stage parasites 16–20 h later. Transfection of schizont stage parasites was done using the protocol described by Hasenkamp and colleagues [63]. Transfected parasites were grown using 2.5 nM WR99210 (a gift from Jacobus Inc.). For the integration via single crossover recombination, transfected parasite lines were cultivated for 14–20 days without WR99210, and then the drug was added again. Normally, after three cycles locus-integrated parasite lines were obtained. These were cloned by limiting dilution [64]. When destabilization domain-expressing parasite lines were used, the small molecule ligand Shield-1 [46] was used at a final concentration of 0.5 μM or 1 μM (stock solution diluted in ethanol) starting directly after transfection of parasites. Shield-1 removal or addition was done in the given time intervals. Experiments were done in biological duplicates or triplicates.

### Plasmid constructs

The following oligonucleotides were used to PCR-amplify the 3' end of the surf4.1 (PlasmoDB ID PF3D7_0402200) reading frame: forward: AGATCTGGAAAATGTAAAATGTTGGAAATTGTATTAG, reverse CTGCAGCTTTTTTTCTTTTATTATTATGTTTATCAAATTCATCC (introduced restriction sites underlined). The amplicons were cloned in pGEM T easy vectors (Promega) and sequenced. Then, the corresponding SURFIN4.1–3'-encoding fragment was excised using Bgl2 and Pst1 and transferred to pRESA-GFP-HA or pRESA-GFP-HA-DD24 vectors [47] digested with the same enzymes. Recombinant plasmids were grown to high quantities using the Maxiprep protocol [65] and used for transfections.

### Real time qPCR

For *surf* transcript quantification, 9 oligo pairs corresponding to the 3D7 *surf* genes available in plasmoDB (version 8) were designed using Primer3 [66] (S1 Table). Notably, *surf* genes 3 and 8 are identical. Whole RNA was purified from synchronized stages (ring stage, directly after sorbitol treatment, trophozoite stage, 20 h after sorbitol treatment, and schizont stage, 30 h after sorbitol treatment) by the Trizol protocol (Life Technologies) and dissolved in pure water. RNAs were then treated with DNAse1 (Fermentas) and cDNA synthesis was done using RevertAid reverse transcriptase (Fermentas) using random hexamer oligos as published earlier [67]. As an endogenous control transcript, the plasmodial serine tRNA ligase transcript (PlasmoDB PF3D7_0717700, herein termed "K1") was used. The primer performance of all surf oligos was compared to PF3D7_0717700 oligos in order to permit relative quantification (Fig C in S1 File).

### Actinomycin treatment

Transfected parasites or untransfected parasites treated or not with Shield-1 were submitted to treatment with Actinomycin D (Sigma) at 20 μg*ml^-1^ for 0, 5 or 30 min as described previously [56]. Total RNAs were purified using Trizol and quantified. The same quantities of RNA were reverse transcribed into cDNA and amplified using qPCR as described above. From obtained Ct values, the 2^-ΔCt^ values for *surf*4.1 were computed and plotted against the time interval of Actinomycin treatment.

### Immunoblotting

For the detection of recombinant proteins in transgenic parasite lines, whole parasite protein extracts were prepared from saponin-lysed IRBCs as described in Methods in Malaria Research [64]. Proteins were loaded on standard discontinuous SDS-polyacrylamide gels and transferred to Hybond C membranes (Amersham). After blocking with 4% skimmed milk in 1xPBS/0.1% Tween20, HA-tagged proteins were recognized using a murine antiHA antibody (Sigma-Aldrich) and afterwards an antiMouse IgG-peroxydase antibody (KPL). Blots were exhaustively washed with PBS/Tween between incubations and finally incubated with WesternPico Super signal substrate (Pierce/Thermo). As a loading control, a murine antiPTEX150 antibody was used. Chemoluminescent signals were captured in an ImageQuant (GE) apparatus and intensities were quantified using ImageJ software (NIH). The obtained values were normalized using the PTEX150 signals.

## Supporting information

S1 File **Figure A: Dynamics of *surf* transcripts in 3D7 cultures grown for 40 reinvasion cycles**. The transcript quantities were measured as described in Methods in three different parasite stages (rings/trophozoites and schizonts, parasite forms similar to the ones shown in Fig 1). **Figure B: Effect of Shield-1 removal and re-addition on *surf* genes other than *surf*4/*surf*4.1**. The transcript quantities were measured by RT-qPCR from schizont stage parasite RNA from the transgenic NF54::pS4-GFP-HA-DD24 line as described.**Figure C: Primer performance of *surf* oligos used throughout the experiments**. Two concentrations of NF54 genomic DNA were used in qPCR experiments as described in Methods and the obtained Ct values of triplicate samples were plotted against the tested locus. In red bars, C_t_ values for 3 ng, in black, C_t_ values for 0.3 ng genomic NF54 DNA.
(PDF)

S1 Table Oligonucleotides used for real time qPCR. Given are the herein used abbreviations and the respective PlasmoDB identity and forward and reverse oligo sequences. Oligos were chosen using Primer3 software [66] on PlasmoDB’s *surf* transcript sequences. The settings for primer selection were: T_m_ 60°C (range 58–62°C), amplicon size 80–120 nt, primer length: 22 nt (range 20–25 nt), minimum GC content of 30%, and default settings for further parameters. See Figure C in S1 File for primer performance on genomic DNA targets.
(DOCX)
