# Supplementary figures and images for: Knockdown of the Plasmodium falciparum SURFIN4.1 antigen leads to an increase of its cognate transcript
Source: PLoS One. 2017 Aug 11;12(8):e0183129. doi: 10.1371/journal.pone.0183129 (PMC5553854; doi:10.1371/journal.pone.0183129)

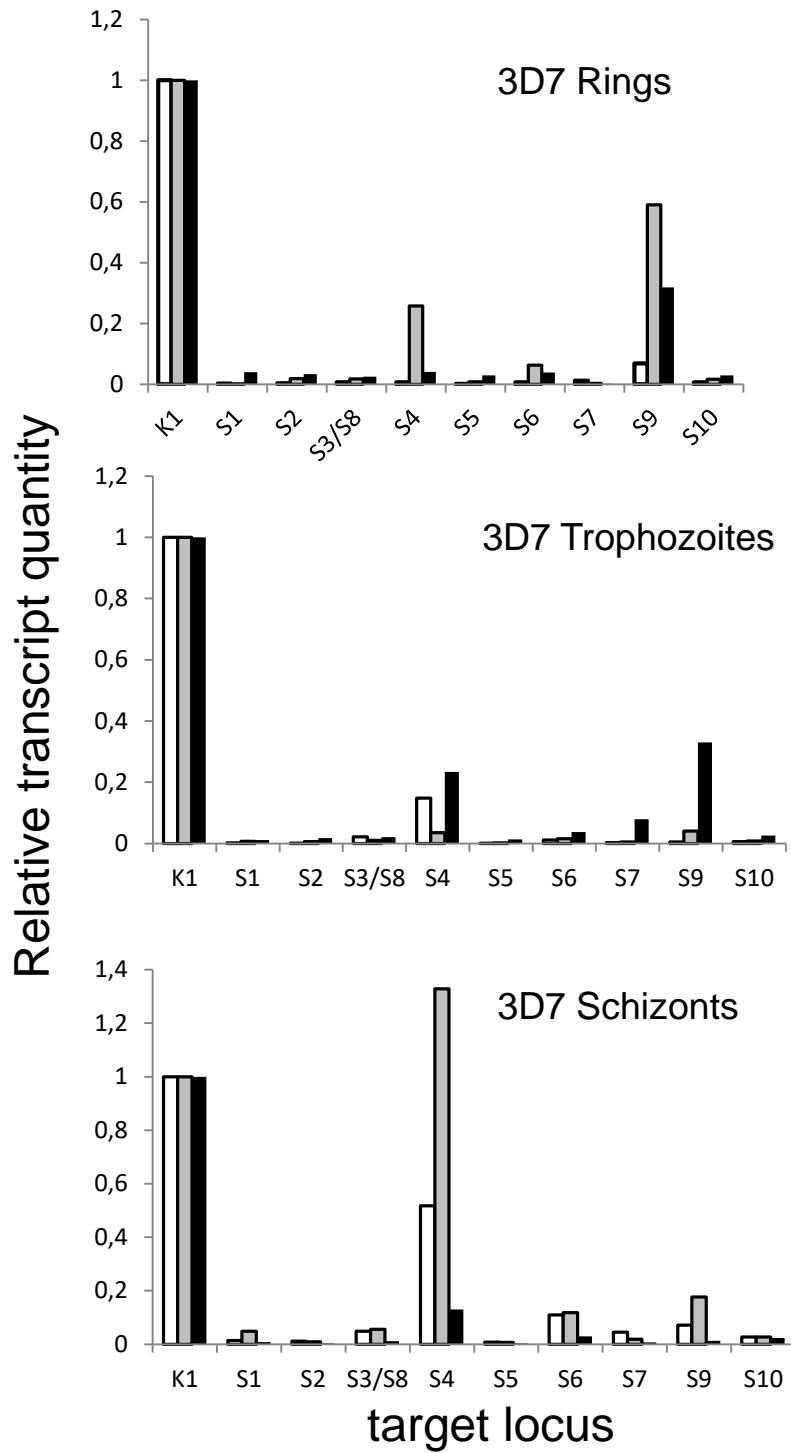

Relative transcript quantity

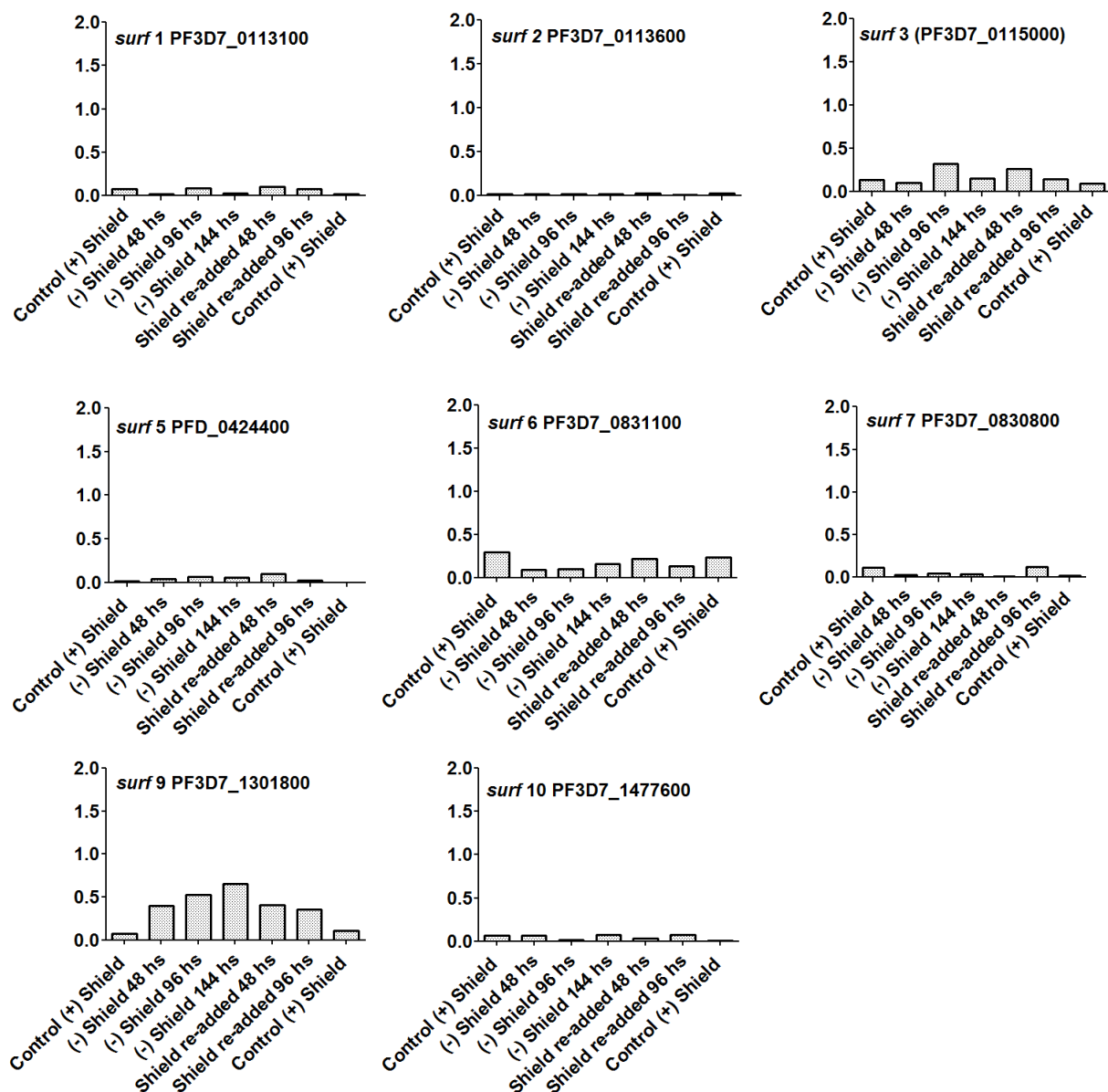

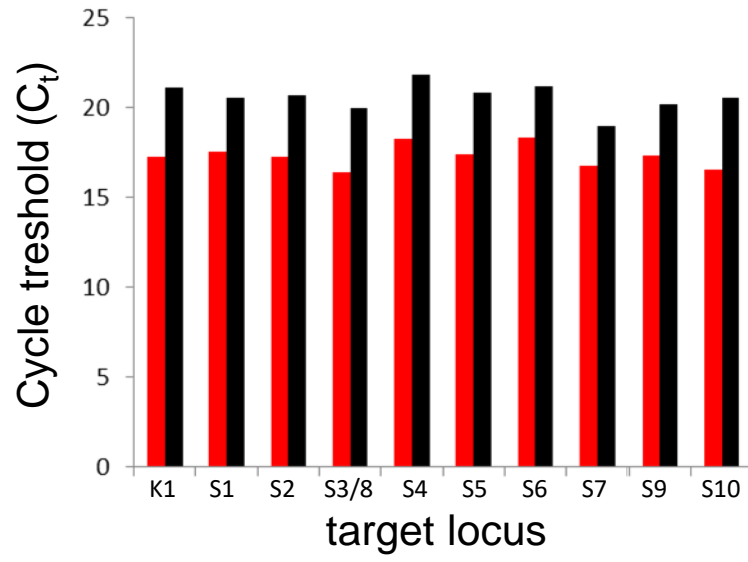

Supplement: S1 File — Figure A: Dynamics of surf transcripts in 3D7 cultures grown for 40 reinvasion cycles. The transcript quantities were measured as described in Methods in three different parasite stages (rings/trophozoites and schizonts, parasite forms similar to the ones shown in Fig 1). Figure B: Effect of Shield-1 removal and re-addition on surf genes other than surf4/surf4.1. The transcript quantities were measured by RT-qPCR from schizont stage parasite RNA from the transgenic NF54::pS4-GFP-HA-DD24 line as described.Figure C: Primer performance of surf oligos used throughout the experiments. Two concentrations of NF54 genomic DNA were used in qPCR experiments as described in Methods and the obtained Ct values of triplicate samples were plotted against the tested locus. In red bars, Ct values for 3 ng, in black, Ct values for 0.3 ng genomic NF54 DNA. (PDF) [file pone.0183129.s001.pdf]
