# Supplementary material for: Knockdown of the Plasmodium falciparum SURFIN4.1 antigen leads to an increase of its cognate transcript
Source: PLoS One. 2017 Aug 11;12(8):e0183129. doi: 10.1371/journal.pone.0183129 (PMC5553854; doi:10.1371/journal.pone.0183129)
Supplement: S1 Table — Given are the herein used abbreviations and the respective PlasmoDB identity and forward and reverse oligo sequences. Oligos were chosen using Primer3 software [66] on PlasmoDB’s surf transcript sequences. The settings for primer selection were: Tm 60°C (range 58–62°C), amplicon size 80–120 nt, primer length: 22 nt (range 20–25 nt), minimum GC content of 30%, and default settings for further parameters. See Figure C in S1 File for primer performance on genomic DNA targets. (DOCX) [file pone.0183129.s002.docx]

**S1 Table**

| ***surf* locus** | **plasmodb.org ID** | **Sequence of forward (F) and reverse (R) oligos for each locus** |
| --- | --- | --- |
| *surf*1 | PF3D7_0113100 | F 5' GAAGAATGGAAAATGACCAAGG |
|  |  | R 5' TCGAAATCGTCCTCTTCGTT |
| *surf*2 | PF3D7_0113600 | F 5' CCCCTGTATCTGAACCGATG |
|  |  | R 5' CGTTTGAGCAAGATGGGAAT |
| *surf*3/8 | PF3D7_0115000 PF3D7_0800700 | F 5' AAAATTCCGGAACAAGACCA |
|  |  | R 5' ATTCCAGATTCCGCATGAAC |
| *surf*4 (surf4.1) | PF3D7_0402200 | F 5' ACACTTGCACAAACAAAACAGG |
|  |  | R 5' CCATCAACTTGCGTTTTCAATA10 |
| *surf*5 (surf4.2) | PF3D7_0424400 | F 5' TCACGAACGCTTGATAGTGC |
|  |  | R 5' TTGCTTTGACCTTCACTTCG |
| *surf*6 | PF3D7_0831100 | F 5' TGCCATTTGATGAACCAGAA |
|  |  | R 5' GCATGTCGTTCAACCCATCT |
| *surf*7 | PF3D7_0830800 | F 5' ACCTCGAGCGATGGAGATAA |
|  |  | R 5' CTCATTGGGTGATCCATCCT |
| *surf*9 | PF3D7_1301800 | F 5' TCCCCTCATTCTTCAGTTCC |
|  |  | R 5' TTTGCAAAGGGTGGAGTAGC |
| *surf*10 | PF3D7_1477600 | F 5' GAAGGGGGTGATAATGGTGA |
|  |  | R 5' TGAACAATGAGGGAAAACCAG |
